# No Clinically Relevant Effect of Heart Rate Increase and Heart Rate Recovery During Exercise on Cardiovascular Disease: A Mendelian Randomization Analysis

**DOI:** 10.3389/fgene.2021.569323

**Published:** 2021-02-18

**Authors:** Josephine Mensah-Kane, Amand F. Schmidt, Aroon D. Hingorani, Chris Finan, Yutang Chen, Stefan van Duijvenboden, Michele Orini, Pier D. Lambiase, Andrew Tinker, Eirini Marouli, Patricia B. Munroe, Julia Ramírez

**Affiliations:** ^1^Clinical Pharmacology, William Harvey Research Institute, Barts and The London School of Medicine and Dentistry, Queen Mary University of London, London, United Kingdom; ^2^Institute of Cardiovascular Science, Faculty of Population Health, University College London, London, United Kingdom; ^3^Department of Cardiology, Division of Heart and Lungs, University Medical Center Utrecht, Utrecht, Netherlands

**Keywords:** heart rate, Mendelian randomization, GWAS, cardiovascular risk, recovery, exercise, UK Biobank

## Abstract

**Background:**

Reduced heart rate (HR) increase (HRI), recovery (HRR), and higher resting HR are associated with cardiovascular (CV) disease, but causal inferences have not been deduced. We investigated causal effects of HRI, HRR, and resting HR on CV risk, all-cause mortality (ACM), atrial fibrillation (AF), coronary artery disease (CAD), and ischemic stroke (IS) using Mendelian Randomization.

**Methods:**

11 variants for HRI, 11 for HRR, and two sets of 46 and 414 variants for resting HR were obtained from four genome-wide association studies (GWASs) on UK Biobank. We performed a lookup on GWASs for CV risk and ACM in UK Biobank (*N* = 375,367, 5.4% cases and *N* = 393,165, 4.4% cases, respectively). For CAD, AF, and IS, we used publicly available summary statistics. We used a random-effects inverse-variance weighted (IVW) method and sensitivity analyses to estimate causality.

**Results:**

IVW showed a nominally significant effect of HRI on CV events (odds ratio [OR] = 1.0012, *P* = 4.11 × 10^–2^) and on CAD and AF. Regarding HRR, IVW was not significant for any outcome. The IVW method indicated statistically significant associations of resting HR with AF (OR = 0.9825, *P* = 9.8 × 10^–6^), supported by all sensitivity analyses, and a nominally significant association with IS (OR = 0.9926, *P* = 9.82 × 10^–3^).

**Conclusion:**

Our findings suggest no strong evidence of an association between HRI and HRR and any outcome and confirm prior work reporting a highly significant effect of resting HR on AF. Future research is required to explore HRI and HRR associations further using more powerful predictors, when available.

## Introduction

Similar to high resting heart rate (HR), a reduced HR increase (HRI) during exercise or a reduced HR recovery (HRR) after exercise is associated with higher cardiovascular (CV) mortality rates (Savonen et al., 2006; Fox et al., 2007; Myers et al., 2007; Arena et al., 2010; Cooney et al., 2010; Zhang et al., 2016). Previous observational studies have reported that a reduced HRI or HRR is a strong predictor of CV events, with odds ratios (ORs) of 1.20 (95% confidence interval [CI] of 1–1.43) and 1.13 (95% CI 1.05–1.21) for each decrement of 10 beats per minute (bpm) (Savonen et al., 2006; Qiu et al., 2017), respectively. However, their causal implication remains to be determined.

Mendelian randomization (MR) is a methodology that uses single-nucleotide variants (SNVs) as instruments to deduce the magnitude and direction of the effect of a risk factor on an outcome, while accounting for potential confounding (Burgess and Thompson, 2015a). MR takes advantage of the fact that alleles are randomly allocated to individuals during meiosis, thus compensating for the effect of confounders on both the risk factor and the outcome. MR studies have revealed that higher resting HR decreases the risk of atrial fibrillation (AF) and ischemic stroke (IS), while increasing all-cause mortality (ACM) risk (Eppinga et al., 2016; Larsson et al., 2019). However, the association between HRI and HRR and CV events has not been evaluated before.

The primary objective of our study was to use two-sample MR to investigate the effect of HRI, HRR, and resting HR on CV events. We also tested their effect on ACM and a range of common subtypes of CV disease, including AF, coronary artery disease (CAD), and IS.

## Materials and Methods

### Selection, Prioritization, and Weighting of Instruments for HRI and HRR

We used 14 SNVs for HRI, and 18 for HRR, reported in the largest published genome-wide association study (GWAS) at *P* < 5 × 10^–8^, including 67,257 individuals from the UK Biobank who participated in an exercise stress test (EST-UKB cohort) (Ramírez et al., 2018) (Supplementary Figure 1). These individuals comprised relatively even numbers of men and women aged 40–69 years at recruitment with European ancestry and no previous CV event (Ramírez et al., 2018). We also included one independent variant for HRI and 3 for HRR from a second GWAS, also from UK Biobank (Supplementary Figure 1) (Verweij et al., 2018). To prevent erroneously inflated type-1 error rates, variants were clumped on a pairwise R-squared of 0.1, both within each of the two traits, and across both datasets. We used the identified SNVs as potential instruments to investigate the causal relationship between HRI and HRR, and CV events, ACM, and CV subtypes. We weighted the instruments by their effect sizes in their respective replication GWASs (Ramírez et al., 2018), which did not include any participants or related individual from the GWAS in which the SNVs were initially discovered, thus avoiding any bias due to population overlap (Supplementary Tables 1,2).

### Selection, Prioritization, and Weighting of Instruments for Resting HR

Three previous publications have reported genetic variants for resting HR (Eppinga et al., 2016; Ramírez et al., 2018; Guo et al., 2019). Eppinga et al. (2016) used a subset of UK Biobank population and identified 64 SNVs reached genome-wide significance (*P* < 5 × 10^–8^). Also using a subset of the UK Biobank population, Ramírez et al. (2018) reported 2 genome-wide significant SNVs independent to these 64 SNVs (Supplementary Figure 1). Finally, Guo et al. (2019) performed a GWAS on resting HR using the available UK Biobank population sample.

We used two different sets of instruments for resting HR (Supplementary Figure 1). For testing with CV events and ACM, we used 66 independent variants from Eppinga et al. (2016) and Ramírez et al. (2018). These instruments were selected to ensure no sample overlap from GWAS sample for CV events and ACM (see below). We weighted the 64 SNVs from Eppinga et al.’s work (Eppinga et al., 2016) by their effect sizes in their replication GWAS, but the two from Ramírez et al. (2018) were weighted by their discovery effect sizes, as there was no replication GWAS (Supplementary Table 3).

For testing with CAD, IS, and AF, we used 458 independent genome-wide significant variants for resting HR, which we weighted by their corresponding effect sizes (Supplementary Table 4). We performed our own GWAS in UK Biobank to identify these variants using 388,237 individuals with pulse rate measurement (as a proxy for heart rate, further details are described in the Supplementary Methods, Supplementary Figure 1), as Guo et al. (2019) did not report the effect sizes for their genetic variants.

### Genetic Associations With the Outcomes

**FIGURE 1:**
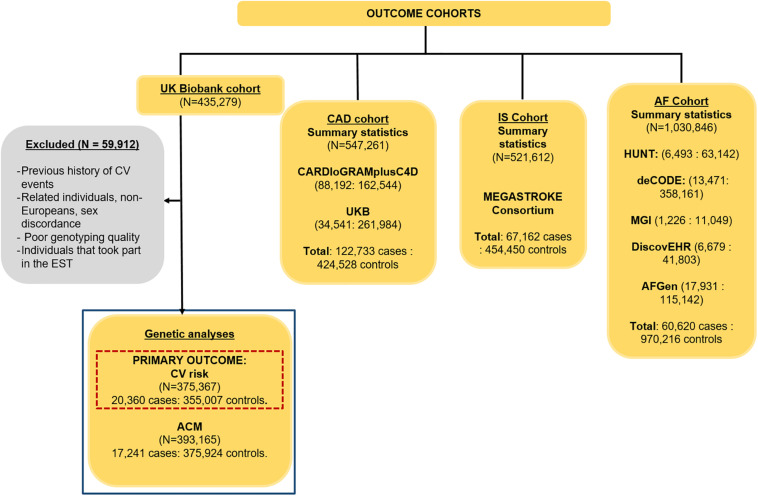
Cohorts used to assess the causality of HRI, HRR, and resting HR on CV events, ACM, CAD, IS, and AF, with their sample sizes and case–control ratios. A GWAS was performed in this project (highlighted in the blue box) for CV events (our primary outcome, highlighted in the dashed red box) and ACM, while the summary statistics were downloaded for CAD (Nelson et al., 2017), IS (Malik et al., 2018), and AF (Nielsen et al., 2018).

The CV events cohort (Figure 1) consisted of 375,367 independent individuals (5.4% cases) from UK Biobank. A CV event was defined as any death or hospital admission due to ischemic heart disease, myocardial infarction, atherosclerosis, cardiomyopathies, cardiovascular disease, coronary artery disease, heart failure, ventricular arrhythmias, and stroke occurring between enrolment in UK Biobank (2006–2008) and March 2017. The specific ICD10 codes are I21, I22, I24, I25, I42, I46–I51, I64, and I70 (detailed ICD10 codes are indicated in Supplementary Table 5). We excluded related, non-European individuals, and subjects with poor genotype and imputed calling, sex discrepancies, and a previous history of a CV event. We only considered incident events, as the participants in the study did not have any underlying known cardiovascular condition. For the definition of events, we used hospital episode statistics and death registration data, both available in UK Biobank. Primary care data was not included.

The ACM cohort consisted of 393,165 (4.4% cases, defined as death from any cause – excluding unnatural causes – up to March 2017) unrelated, European individuals with good genotype and imputed calling, and no sex discrepancies from UKB. We also excluded from both the CV and ACM cohorts any individual who were part of the EST-UKB cohort and conducted our own GWASs for the primary outcome, CV events, as well as for ACM. Details of the GWAS methodology used for both outcomes can be found in the Supplementary Methods. Population characteristics for the individuals in the FULL-UKB cohort were determined, and the Wilcoxon unpaired test (for continuous variables) and Fisher test (for binary variables) were used to assess significant differences in covariates between cases and controls.

Genetic associations with CAD, IS, and AF were obtained from publicly available GWAS summary statistics from various large consortia (Figure 1). Summary statistics for CAD were obtained from a meta-analysis of 547,261 individuals (122,733 cases, 22.4%) from the Coronary Artery Disease Genome-Wide Replication and Meta-Analysis plus The Coronary Artery Disease Consortium (CARDIoGRAMplusC4D), as well as 296,525 individuals from UK Biobank (Figure 1) (Nelson et al., 2017). The IS summary statistics were obtained from 521,612 individuals (67,162 cases, 12.9%) from the MEGASTROKE consortium (Figure 1, IS) (Malik et al., 2018). AF summary statistics combined results from *N* = 1,030,846 (60,620 cases, 5.9%) included in a meta-analysis of five different studies: the HUNT cohort, a Norwegian population-based cohort of ∼125,000 individuals; the deCODE study, comprised of data from 10,269 European individuals aged from 30 to 89; the Montreal Heart Institute Biobank, a hospital-based cohort currently comprised of ∼17,000 French-Canadians; the DiscovEHR cohort of 50,726 adults of 95.5% European ancestry; and the AF Genetics (AFGen) consortium (Figure 1) (Nielsen et al., 2018). Details of the definitions for CAD, IS, and AF can be found in their respective studies (Nelson et al., 2017; Malik et al., 2018; Nielsen et al., 2018).

**FIGURE 2:**
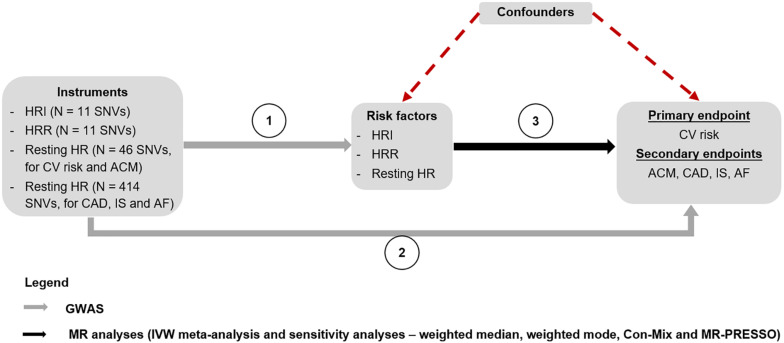
MR study design. The genetic associations of the instruments for HRI, HRR, and resting HR were derived from the GWASs performed by Eppinga et al. (2016), Ramírez et al. (2018), Verweij et al. (2018), and an internal GWAS on resting HR using the entire UK Biobank population, respectively (Step 1). A lookup of these instruments was performed in GWASs for CV events and ACM conducted in the UK Biobank population, to find the genetic associations with these two outcomes. The genetic associations of the instruments with CAD (Nelson et al., 2017), IS (Malik et al., 2018), and AF (Nielsen et al., 2018) were derived by conducting a lookup of the SNVs in the respective GWAS summary statistics for each outcome (Step 2). Finally, MR analyses were conducted to estimate the causal associations between HRI, HRR, and resting HR, and each of the outcomes using an IVW meta-analysis, as well as sensitivity analyses (Step 3). ACM, all-cause mortality; AF, atrial fibrillation; CAD, coronary artery disease; Con-Mix, contamination mixture; CV, cardiovascular; GWAS, genome-wide association study; HR, heart rate; HRI, heart rate increase; HRR, heart rate recovery; IS, ischemic stroke; IVW, inverse variance weighted; MR, Mendelian randomization; SNV, single-nucleotide variant.

A lookup of all instruments for HRI, HRR, and resting HR were conducted in the GWASs we performed for CV events and ACM, as well as in the summary statistics for CAD, AF, and IS, to retrieve genetic associations with the outcomes (Figure 2).

### MR Analyses

We used a random-effects inverse-variance-weighted (IVW) MR estimator^1^ to derive ORs for each outcome per bpm increase in HRI, HRR, and resting HR (Figure 2). All analyses were conducted in R version 3.5.1, using the R package “TwoSampleMR,” which was used in the harmonization of instruments to ensure consistency in the direction of association (Hemani et al., 2018). One palindromic SNV for HRI, two out of 66 from the first set of variants for resting HR, and 19 out of 458 from the second set of variants for resting HR with intermediate frequencies (i.e., close to 0.5) were excluded from the MR analyses to prevent harmonization errors (Supplementary Tables 1, 3, 4).

The two most important assumptions in MR are that (i) the instruments need to be truly associated with the corresponding risk factor (HRI, HRR, or resting HR) and (ii) the effect of the instruments on the corresponding outcome needs to be mediated by the risk factor only. This means that weak and pleiotropic instruments should be avoided as they can strongly bias the effect sizes. We first assessed the strength of all instruments based on the *F* statistics (calculated as $F=\;{\text{β}}_{exposure}^{2}/SE_{exposure}^{2}$) (Kus et al., 2020), which indicated that 3, 10, 18, and 25 instruments for HRI, HRR, and both sets of resting HR instruments, respectively, had *F* < 10 (Burgess et al., 2013) (Supplementary Tables 1–4). This led to a total of 11 strong instruments for HRI, 11 for HRR, and 46 and 414 for both sets of instruments for resting HR used in the MR analyses, explaining a percentage variance of 1.12, 0.75, 2.63, and 9.10%, respectively (Burgess and Thompson, 2015b). We next addressed the second assumption by performing sensitivity analyses. We compared the results obtained using the IVW method with those from the weighted median (Bowden et al., 2016), weighted mode (Hartwig et al., 2017), contamination mixture (Con-Mix) (Burgess et al., 2020), and MR-PRESSO (Verbanck et al., 2018), as these methods can provide valid MR effect sizes under the presence of horizontal pleiotropy. For the Con-Mix method, we used the default value of the psi parameter, which was 0, corresponding to 1.5 times the standard deviation of the ratio estimates.

For any significant associations between any of the risk factors and any of the outcomes, we tested the reverse association (i.e., from the outcome to the risk factor) using the same methods described here. To test if HRR (HRI) was mediating the association of HRI (HRR) with each outcome, we ran multivariable MR (Burgess and Thompson, 2015b), which provides an estimate of the direct effect of HRI (HRR) on each outcome after adjusting for HRR (HRI) as a potential mediator in the causal pathway. We then repeated multivariable MR by further adjusting for resting HR. Finally, we investigated whether any potential confounders might lie in the causal pathway by running multivariable MR (Burgess and Thompson, 2015b) to estimate the direct effect of resting HR on AF (which is the only consistent association, as shown in section “Results”) after adjusting for potential confounders. In particular, body mass index (BMI) and diabetes are two important risk factors for AF (Schnabel et al., 2009; Chamberlain et al., 2011), and previous findings have reported on the genetic correlation between resting HR and these risk factors (Guo et al., 2019; Munroe Patricia et al., 2019), suggesting that there is a risk for pleiotropy. We downloaded summary statistics for BMI and diabetes from the GWAS catalog^2^ from two recent GWAS (Scott et al., 2017; Pulit et al., 2019). We identified 325 and 40 sentinel SNVs (*P* < 5 × 10^–8^) for each risk factor, respectively. After excluding those that were within a 1-Mb window around the 458 sentinel SNVs for resting HR and those in linkage disequilibrium (*r*^2^ > 0.1), a total of 747 independent SNVs were used as instrumental variables for the multivariable MR analyses.

To control for false-positive findings due to multiple testing, we applied a conservative Bonferroni-corrected significance threshold, adjusted for the number of risk factors and endpoints (*P* = 0.05/(3 × 5) = 3.3 × 10^–3^). A *P*-value <0.05 was considered evidence for nominal significance.

To estimate the power of our study, we used a non-centrality parameter-based approach (Brion et al., 2012), implemented in a publicly available mRnd web tool^3^. As all outcomes were binary, we calculated minimal ORs of the outcome variable per SD of the risk factor variable that was detectable (power = 0.8, α = 0.05) in our study. The results of power calculations are provided in Supplementary Table 6 and indicate that we would have 80% power to detect strong effect sizes of HRI on CAD and AF, of HRR on AF, and of resting HR on IS and AF, but more power would be needed to detect smaller effects on the remaining outcomes.

## Results

### CV and ACM GWAS Population Characteristics

The baseline characteristics of the 375,367 individuals in the CV events GWAS from the Full-UKB cohort are indicated in Table 1. The 393,165 individuals included in the GWAS for ACM had similar characteristics, as they were also part of the UK Biobank study. During the follow-up, 20,360 individuals experienced a CV event, and 17,241 had ACM (Figure 1). All the covariates that were significantly different across case and control groups (Table 1) were used as covariates in our GWASs for CV events and ACM.

**TABLE 1:** Baseline characteristics of UKB individuals studied in the GWAS for CV risk.

Characteristic	All Participants	CV risk diagnosis	No CV risk diagnosis	*P*-value
	(*N* = 375,367)	(*N* = 20,360)	(*N* = 355,007)	
Male (%)	44.2	63.9	43	<2.2e-16
Age at recruitment (years)	56.6 (8.0)	61.1 (6.5)	56.3 (8.0)	<2.2e-16
BMI (kg/m^2^)	27.4 (4.8)	28.9 (5.2)	27.3 (4.8)	<2.2e-16
SBP (mmHg)	138.3 (18.9)	144.4 (19.5)	137.9 (18.8)	<2.2e-16
Diabetes status (%)	4.4	11.9	4	<2.2e-16
Cholesterol Status (%)	11.3	22.8	10.6	<2.2e-16

### Genetic Associations With Endpoints

Heritability estimations, after conversion to the liability scale, were 6.8 and 2.74% for CV events and ACM respectively. QQ plots and Manhattan plots for both GWASs are shown in Supplementary Figures 2 and 3.

A lookup of all SNVs included as instruments for HRI, HRR, and resting HR in our GWASs for CV events and ACM, and in the summary statistics for CAD, AF, and IS can be found in Supplementary Tables 1–3, respectively.

### MR Analyses

**FIGURE 3:**
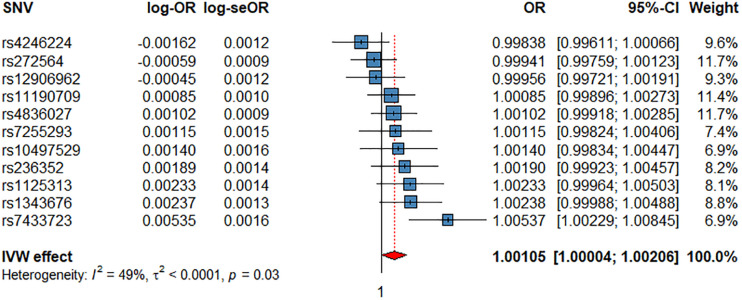
Associations of 1 genetically predicted increase in beats per minute of HRI with CV events. SNV, single-nucleotide polymorphism; OR, odds ratio; se, standard error; CI, confidence interval; l2, heterogeneity statistic l^2^; τ^2^, between-SNV variance.

The IVW showed evidence of a nominal association between HRI and CV events (OR = 1.0012, *P* = 4.11 × 10^–2^, Figure 3, Table 2), indicating that for each increment of 1 bpm in HRI the risk for CV events increases by 0.2%. However, sensitivity analyses suggested that there was no significant effect after adjusting for horizontal pleiotropy (Table 2). Even though the IVW, the weighted median, mode, or MR-PRESSO did not indicate a significant effect of HRI on CAD or AF (Supplementary Figures 4, 5 and Table 2), the Con-Mix method showed a nominally significant association (OR = 0.9802, *P* = 6.92 × 10^–3^ for CAD, and OR = 0.9608, *P* = 1.25 × 10^–2^, Table 2). There was no evidence for causality between HRI and ACM or IS (Supplementary Figures 6, 7 and Table 2).

**TABLE 2:** Causal associations between HRI, HRR and resting HR, and CV, ACM, CAD, IS and AF risk using Mendelian randomization.

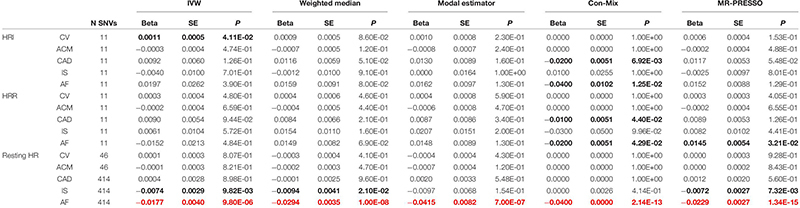

*ACM, all-cause mortality; AF, atrial fibrillation; Beta, effect size (logarithm of odds ratio per 1 beat per minute increase in HRI, HRR and resting HR, respectively); CAD, coronary artery disease; Con-Mix, contamination mixture estimator; CV, cardiovascular; HR, heart rate; HRI, heart rate increase; HRR, heart rate recovery; IS, isquemic stroke; IVW, inverse-variance weighted method; SE, standard error; SNV, single-nucleotide polymorphism.*

*Nominally significant causal associations (*P* < 0.05) are indicated in bold.*

*Bonferroni-corrected significant causal associations (*P* < 0.0033) are indicated in red.*

*Genetic associations with CV and ACM were obtained from a GWAS performed in this manuscript, whereas genetic associations with CAD, IS and AF were taken from published studies (PMID 28714975, 29531354, and 30061737, respectively).*

**FIGURE 4:**
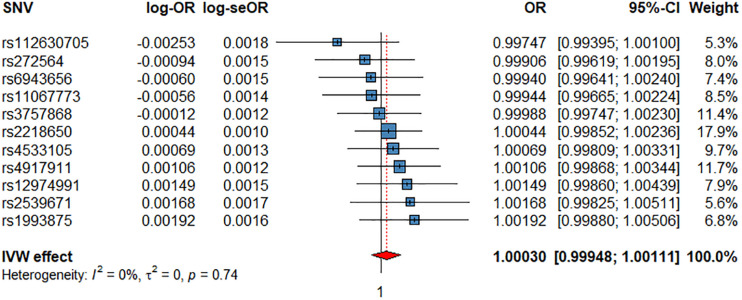
Associations of 1 genetically predicted increase in beats per minute of HRR with CV events. SNV, single-nucleotide polymorphism; OR, odds ratio; se, standard error; CI, confidence interval; l2, heterogeneity statistic l^2^; τ^2^, between-SNV variance.

We did not find evidence for an effect of HRR on CV events (Figure 4), ACM, or any CV subtype (Supplementary Figures 8–11 and Table 2). However, the Con-Mix method suggested a nominally significant effect of HRR on CAD (OR = 0.9900, *P* = 4.40 × 10^–2^), and on AF (OR = 0.9802, *P* = 4.29 × 10^–2^), which was also supported by MR-PRESSO, but in an opposite direction (OR = 1.0146, *P* = 3.21 × 10^–2^, Table 2).

The IVW meta-analysis found a significant effect of resting HR on AF (OR = 0.9825, *P* = 9.80 × 10^–6^, Supplementary Figure 12 and Table 2), which was supported by all sensitivity analyses (Table 2). In addition, the IVW indicated a nominally significant association with IS (OR = 0.9926, *P* = 9.82 × 10^–3^, Supplementary Figure 13), which was supported by the weighted median (OR = 0.9906, *P* = 2.10 × 10^–2^) and MR-PRESSO (OR = 0.9928, *P* = 7.32 × 10^–3^, Table 2). No association was found for CV, ACM, or CAD (Supplementary Figures 14–16 and Table 2).

The association between resting HR and AF was the only consistent significant association among all MR analyses conducted; thus, we tested for any reverse effect by assessing the effects of genetically predetermined AF risk on resting HR. For this, we used 111 independent genetic variants associated with AF at a genome-wide significant level (*P* < 5 × 10^–8^) (Nielsen et al., 2018). We found neither the IVW method nor any sensitivity MR analysis showed significant estimates.

Multivariable MR suggested nominally significant direct associations of HRI on CV events (OR = 1.0010, *P* = 4.78 × 10^–2^) and CAD (OR = 1.0101, *P* = 2.16 × 10^–2^) after adjusting for HRR (Supplementary Table 7a). Similarly, there was a nominally significant direct effect of HRR on CAD after adjusting for HRI (OR = 1.0101, *P* = 2.05 × 10^–2^, Supplementary Table 7a). When resting HR was also adjusted for in the model as a mediator, no associations were statistically significant (Supplementary Table 7b).

As association between resting HR and AF was the only consistent significant association among all MR analyses conducted, we ran multivariable MR to investigate the direct effect after adjustment for BMI and diabetes. Results indicated that a resting HR remains significantly (and inversely) associated with AF (OR = 0.987, *P* = 8.38 × 10^–5^) after adjusting for both BMI (OR = 1.301, *P* = 8.80 × 10^–8^) and diabetes (OR = 1.01, *P* = 5.39 × 10^–1^, Supplementary Table 7c).

## Discussion

In this study, we tested, for the first time, for a potential causal effect of HRI and HRR on CV events and ACM in UK Biobank and three CV subtypes (AF, CAD, and IS) in various different large GWAS consortia. We additionally tested for potential causal effects between resting HR and the same outcomes. Our main findings indicate there was no strong evidence for a significant association between HRI, HRR, and any outcome, but we identified a significant association between lower resting HR and AF risk.

The IVW method indicated a nominally significant effect of HRI on CV events, but this effect was much less extreme and in an opposite direction than observational associations reported in literature (Gulati et al., 2005; Savonen et al., 2006; Myers et al., 2007), and also none of the sensitivity methods showed significant results (Table 2). The observation that none of the sensitivity analyses supported the IVW association indicates it is biased by horizontal pleiotropy, i.e., there are other unaccounted risk factors that are in the causal pathway between HRI and CV risk. When adjusting for HRR as a mediator in multivariable MR, HRI maintained a nominally significant association with CV events (Supplementary Table 7a). However, this direct association became non-significant after further adjusting for resting HR in the multivariate model (Supplementary Table 7b). Approximately 85% of the CV events in the CV GWAS were CAD, so the MR analysis on CAD can be viewed as an external validation for the interpretation of our results for CV events. The CAD dataset (*N* = 547,261) was comprised of 22% cases (Figure 1) and thus offered more power for the MR analyses than UK Biobank, which had a lower sample size and only 5.42% cases with CV events (Figure 1, Supplementary Table 6). The results indicating HRI was not causally associated with CAD (Supplementary Figure 4 and Table 2) further suggests that the IVW association observed between HRI and CV events should be interpreted with caution.

Regarding HRR, we did not observe a significant association with any of the endpoints. Con-Mix and multivariable MR (when adjusting for HRI) suggested a nominally significant association between HRR and CAD, and Con-Mix and MR-PRESSO also indicated a nominally significant association between HRR and AF (Table 2 and Supplementary Tables 7a,b). However, the direction of effect was inconsistent, the statistical significance of the associations was weak (i.e., the *P*-value was only nominally significant), and there was lack of support from other MR methods, potentially indicating no strong evidence for an effect of HRR on CV events, AF, CAD, IS, or ACM. However, although we have used instruments from the largest available GWAS on this risk factor, their percentage variance explained was low (0.75%); therefore, our interpretation of results should be taken with caution.

Although previous observational studies reported that both HRI and HRR are significantly associated with CV events (Savonen et al., 2006; Qiu et al., 2017), we did not find strong evidence for any association. A possible explanation could be that observational studies do not entirely account for all potential confounders. For example, reduced cardiorespiratory fitness, chronic inflammation, and insulin resistance can lead to a reduced HRI, as well as to higher CV risk (Fletcher et al., 2001; Stewart, 2004). In addition, individuals with reduced HRI are also prone to suffer from metabolic risk factors, such as obesity and dyslipidemia, and to engage in unhealthy lifestyle behaviors like smoking.

The IVW and all four sensitivity analyses that account for horizontal pleiotropy supported a significant inverse association between resting HR and AF risk, supporting previous results by Larsson et al. (2019). When comparing the IVW and weighted median estimates, they reported ORs of 0.82 (*P* = 0.60 × 10^–2^) and 0.73 (*P* = 2.9 × 10^–7^), respectively, per 10-bpm increase in resting HR, whereas our estimates are 0.84 (*P* = 9.80 × 10^–6^) and 0.75 (*P* = 1 × 10^–8^), respectively, indicating more precise estimates. The main differences across both studies are that they used the 64 SNVs in our first set of instruments for resting HR (Eppinga et al., 2016) in their MR analysis, whereas we used a much larger set of instruments (414), explaining four times more percentage variance than the 64 SNVs (9.10% versus 2.64%). In addition, they used the summary statistics from the Atrial Fibrillation Consortium (*N* = 537,409) (Roselli et al., 2018), which included UK Biobank, and we used summary statistics from the largest meta-analysis on AF, excluding UK Biobank (*N* = 1,030,846, Figure 1).

Larsson et al. (2019) also reported an inverse association between resting HR and IS using the IVW method, but it became non-significant when using sensitivity analyses that account for horizontal pleiotropy. In our study, we found nominally significant results from the IVW, weighted median, and MR-PRESSO methods, but the weak significance could confirm the presence of horizontal pleiotropy. Finally, our findings of no effect of resting HR on CAD are consistent with results by Larsson et al. (2019). Regarding ACM, our results indicating no effect were consistent with those reported by Eppinga et al. (2016), in which they reported a non-significant association when only genome-wide significant variants were considered (using the same instruments as we did) in their IVW meta-analysis. Of note, when they included SNVs that were not genome-wide significant, their association between resting HR and ACM was significant. Whether our results would change if we had included non-genome-wide significant SNVs remains unclear, but it is out of the scope of this manuscript.

Major strengths of our investigation include the large sample sizes used, for example, in the single UK Biobank cohort, 375,367 individuals were studied for CV events and 393,165 for ACM (Figure 1). Also, by incorporating summary-level genetic data from various large consortia, including more than 1 million individuals in the AF study, over half a million individuals in the CAD study, and just under half a million individuals in the IS study (Figure 1), our analyses used the largest and most recent meta-analyses. For instruments, we combined SNVs from the only two studies that have shown genome-wide significance with HRI and HRR and thus used the largest number of SNVs available for these risk factors in GWAS literature (Ramírez et al., 2018; Verweij et al., 2018). Regarding resting HR, we used the best possible instruments for each outcome to avoid sample overlap. In addition, we strived to satisfy the main MR assumptions by excluding SNVs with *F* < 10 (Burgess et al., 2013) and by performing sensitivity analyses that account for potential horizontal pleiotropy.

There were some limitations in our study. Although we used results from the largest available GWAS for all risk factors and outcomes, more power would be needed to identify small effects on CV risk and ACM (Supplementary Table 6), so our findings need to be interpreted with caution and validated after they can be repeated when additional data becomes available. Although we avoided any bias due to population overlap in our analyses with CV events and ACM, there was population overlap in our CAD analysis, as the CAD cohort included individuals from UKB (*N* = 296,525, Figure 1), which is likely to overlap with individuals in the EST-UKB cohort, leading to bias in the causal effect sizes towards the null hypothesis. We also restricted our samples to European, middle-aged to elderly, relatively healthy individuals with no previous history of a CV event; therefore, the results in other ethnic groups remain unclear. Moreover, for the instruments for HRI and HRR, effect sizes for the SNVs reported by Ramírez et al. (2018) were derived from their replication sample, thus avoiding population bias, but the same approach could not be applied for Ramirez et al. resting HR instruments, as this GWAS did not have a validation dataset for a replication GWAS. Also, the participants in the risk factor GWASs were healthier than those in the outcome GWASs; in particular, participants in the IS and AF GWASs were older and therefore the association results could be affected by selection bias (Gkatzionis and Burgess, 2018). Finally, it should be noted that both HRI and HRR were measured from submaximal workloads in the UK Biobank GWAS (Ramírez et al., 2018; Verweij et al., 2018), while maximal workloads have shown to be superior in predicting CV events and ACM (Qiu et al., 2017). Therefore, participants did not reach their maximum HR during exercise, limiting the quantitative range of HRI and HRR, the number of potentially associated variants, and the explained variance. Future studies assessing the genetic contribution of both HRI and HRR using maximal workloads are needed to confirm our findings.

## Conclusion

In conclusion, we found nominal evidence for an effect of HRI on CV events, although the estimation was much lower, and in an opposite direction, than that from observational studies. We found no evidence for an effect of HRR on CV events, ACM, or any CV subtype. We also confirm prior work supporting resting HR to be inversely associated with AF risk.

## Data Availability Statement

The original contributions presented in the study are included in the article/Supplementary Material, further inquiries can be directed to the corresponding author/s.

## Author Contributions

JM-K contributed to the conceptualization, data curation, formal analysis, investigation, methodology, resources, software, validation, visualization, and writing, reviewing, and editing the original draft. SVD contributed to data analysis, resources, and writing, reviewing, and editing the draft. YC contributed to the data curation, formal analysis, investigation, methodology, visualization, and writing the draft. AFS, ADH, CF, and EM contributed to the methodology, and reviewing and editing the draft. MO, PDL, and AT contributed to the conceptualization, funding acquisition, investigation, methodology, project administration, and reviewing and editing the draft. JR and PBM contributed to the conceptualization, formal analysis, funding acquisition, investigation, methodology, project administration, supervision, and writing, reviewing, and editing the original draft.

## Conflict of Interest

AS has received unrestricted funding from Servier. The remaining authors declare that the research was conducted in the absence of any commercial or financial relationships that could be construed as a potential conflict of interest.

## Footnotes

1 https://www.rdocumentation.org/packages/meta/versions/4.9-6/topics/metagen

2 https://www.ebi.ac.uk/gwas/

3 http://cnsgenomics.com/shiny/mRnd/
