# Supplementary material for: No Clinically Relevant Effect of Heart Rate Increase and Heart Rate Recovery During Exercise on Cardiovascular Disease: A Mendelian Randomization Analysis
Source: Front Genet. 2021 Feb 18;12:569323. doi: 10.3389/fgene.2021.569323 (PMC7931909; doi:10.3389/fgene.2021.569323)
Supplement: Supplementary file 1 [file Data_Sheet_1.docx]

**Supplementary Methods**

**GWAS for resting HR**

Pulse rate in UK Biobank (data-field 102) was used as the phenotype of resting HR for the association analysis. In total, 475,167 individuals participated in the initial assessment for pulse rate during 2006-2010, and three repeat assessments were conducted with fewer participants during 2012-2019. If the pulse rate of an individual was available at the first assessment, then data from the first assessment were used. If no pulse rate record at the first assessment, then data from the next available assessment were used. For each assessment, two replicate measurements of pulse rate were recorded. If both replicate measurements were available, an average pulse rate were calculated and if not, the available measurement was taken for analysis. Next, we applied quality control to the extracted pulse rate data. Participants of non-European ancestry, participants without pulse rate record, and participants with extreme pulse rate record (< 40 or > 120 bpm) and cardiovascular problems were excluded from the cohort. Furthermore, participants using beta-blockers were removed.

First, markers genotyped by two chip arrays in UK Biobank were selected for 388,237 individuals. Next, quality control was applied to these markers by using package PLINK 1.9 [1] with the following filters: minor allele frequency (MAF) >= 0.05, Hardy-Weinberg equilibrium (HWE) test with p value < 10^-6^ and missingness < 0.015. In addition, imputed markers from UK Biobank (version 3) were employed for association studies. Quality control for imputed markers was performed while running GWAS using package BOLT-LMM[2] with filters including minor allele frequency >= 0.01 and imputation quality INFO > 0.3.

A mixed-effect model provided by package BOLT-LMM [2] was adopted to examine the association between 9,771,482 imputed genetic variants and resting HR for the 388,237 individuals. Covariates, including age, age squared, sex, BMI, a binary variable indicating two genotyping arrays and the first 10 principle components, were added into the model to control false positives. A P value of 5×10^-8^ was used as the genome-wide significance level.

After the GWAS, the associated loci were identified by defining a 1 Mb genomic region including 500 kb at either side of the lead variant. If the genomic distance between two lead variants was less than 1 Mb, two loci were merged as one locus (the size of the new locus is greater than 1 Mb). Next, a model selection method (--cojo-slct) in GCTA [3] package (conditional analysis) was used to detect secondary independent association signals for associated loci of resting HR. We used the 1000G reference panel [4] as the required linkage disequilibrium reference panel and the default argument of some options, such as MAF (--maf 0.01), regional significant association p-value threshold (--cojo-p 5e-8) and the distance of complete linkage equilibrium (--cojo-wind 10000) within the --cojo-slct option was kept. If the frequency difference of the distinct association signal between GWAS samples and the reference panel was greater than 0.1, then this variant was dropped and --cojo-slct analysis would be repeated again until no signals with the frequency difference greater than 0.1.

**GWAS for CV risk and ACM**

Model SNVs (with missingness <0.0015, a Hardy-Weinberg equilibrium, P-value threshold of 1 x 10^−6^ and a minor allele frequency > 0.05) were selected from the genotyped SNVs for the GWASs using PLINK software version 1.9 [1].

Then, with these model SNVs, the proportion of the variance in CV risk and ACM explained by genetics (i.e. heritability) was calculated using BOLT-REML[5]. As the traits being analysed were case-control, the observed-scale heritability estimates were converted to the liability-scale[6].

Finally, with the converted heritability estimates, a GWAS was conducted for CV risk and ACM, using BOLT-LMM [2], which can also be used for case-control phenotypes ((https://alkesgroup.broadinstitute.org/BOLT-LMM/downloads/BOLT-LMM_v2.3.4_manual.pdf).

The confounding variables accounted for in the regression model were those showing to be significantly different across the case and control groups in the individuals studied for both outcomes, i.e. sex, age, diabetes mellitus, hypercholesterolemia, systolic blood pressure, body mass index, as well as the genetic array used for the genotype calling (Table 1).

**Supplementary References**

[1] C.C. Chang, C.C. Chow, L.C. Tellier, S. Vattikuti, S.M. Purcell, and J.J. Lee, Second-generation PLINK: rising to the challenge of larger and richer datasets. GigaScience 4 (2015).

[2] P.-R. Loh, G. Tucker, B.K. Bulik-Sullivan, B.J. Vilhjálmsson, H.K. Finucane, R.M. Salem, D.I. Chasman, P.M. Ridker, B.M. Neale, B. Berger, N. Patterson, and A.L. Price, Efficient Bayesian mixed-model analysis increases association power in large cohorts. Nature Genetics 47 (2015) 284-290.

[3] J. Yang, S.H. Lee, M.E. Goddard, and P.M. Visscher, GCTA: A Tool for Genome-wide Complex Trait Analysis. The American Journal of Human Genetics 88 (2011) 76-82.

[4] A. Auton, G.R. Abecasis, D.M. Altshuler, R.M. Durbin, G.R. Abecasis, D.R. Bentley, A. Chakravarti, A.G. Clark, P. Donnelly, E.E. Eichler, P. Flicek, S.B. Gabriel, R.A. Gibbs, E.D. Green, M.E. Hurles, B.M. Knoppers, J.O. Korbel, E.S. Lander, C. Lee, H. Lehrach, E.R. Mardis, G.T. Marth, G.A. McVean, D.A. Nickerson, J.P. Schmidt, S.T. Sherry, J. Wang, R.K. Wilson, R.A. Gibbs, E. Boerwinkle, H. Doddapaneni, Y. Han, V. Korchina, C. Kovar, S. Lee, D. Muzny, J.G. Reid, Y. Zhu, J. Wang, Y. Chang, Q. Feng, X. Fang, X. Guo, M. Jian, H. Jiang, X. Jin, T. Lan, G. Li, J. Li, Y. Li, S. Liu, X. Liu, Y. Lu, X. Ma, M. Tang, B. Wang, G. Wang, H. Wu, R. Wu, X. Xu, Y. Yin, D. Zhang, W. Zhang, J. Zhao, M. Zhao, X. Zheng, E.S. Lander, D.M. Altshuler, S.B. Gabriel, N. Gupta, N. Gharani, L.H. Toji, N.P. Gerry, A.M. Resch, P. Flicek, J. Barker, L. Clarke, L. Gil, S.E. Hunt, G. Kelman, E. Kulesha, R. Leinonen, W.M. McLaren, R. Radhakrishnan, A. Roa, D. Smirnov, R.E. Smith, I. Streeter, A. Thormann, I. Toneva, B. Vaughan, X. Zheng-Bradley, D.R. Bentley, R. Grocock, S. Humphray, T. James, Z. Kingsbury, H. Lehrach, R. Sudbrak, M.W. Albrecht, et al., A global reference for human genetic variation. Nature 526 (2015) 68-74.

[5] P.-R. Loh, G. Bhatia, A. Gusev, H.K. Finucane, B.K. Bulik-Sullivan, S.J. Pollack, T.R. de Candia, S.H. Lee, N.R. Wray, K.S. Kendler, M.C. O'Donovan, B.M. Neale, N. Patterson, A.L. Price, and C. Schizophrenia Working Group of the Psychiatric Genomics, Contrasting genetic architectures of schizophrenia and other complex diseases using fast variance-components analysis. Nature Genetics 47 (2015) 1385-1392.

[6] Sang H. Lee, Naomi R. Wray, Michael E. Goddard, and Peter M. Visscher, Estimating Missing Heritability for Disease from Genome-wide Association Studies. The American Journal of Human Genetics 88 (2011) 294-305.
