# Supplementary material for: No Clinically Relevant Effect of Heart Rate Increase and Heart Rate Recovery During Exercise on Cardiovascular Disease: A Mendelian Randomization Analysis
Source: Front Genet. 2021 Feb 18;12:569323. doi: 10.3389/fgene.2021.569323 (PMC7931909; doi:10.3389/fgene.2021.569323)
Supplement: Supplementary file 2 [file Image_1.pdf]

Supplementary Figures

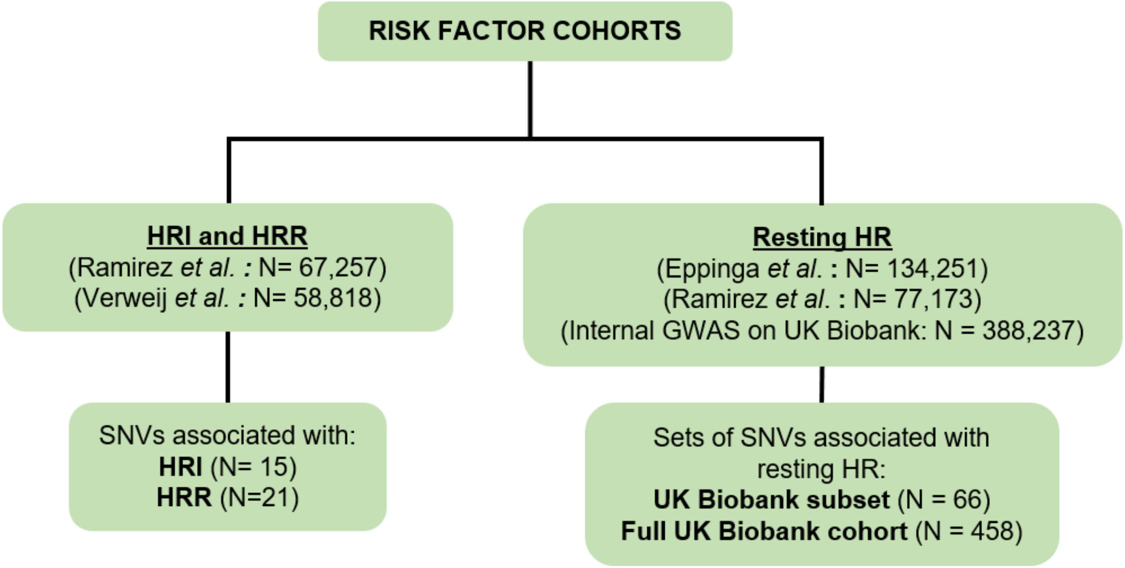

Supplementary Figure 1: Cohorts, reported studies and genome-wide association studies used to derive the genetic variants associated with the risk factors, HRI, HRR and resting HR, including their sample sizes.

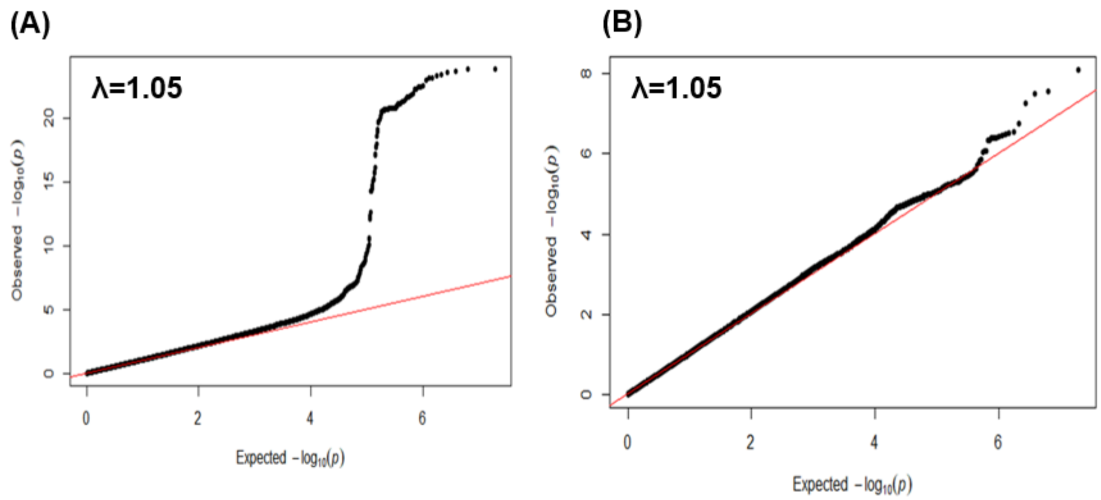

Supplementary Figure 2: QQ plots for GWAS for CV risk (a) and ACM (b)

QQ plots showing the negative logarithm of the observed P-values (y axis) against expected P-values (x axis) from a normal distribution. Each black dot represents the P-value for each SNV, and the red line represents the null hypothesis of no significant association between the SNVs and the outcome.

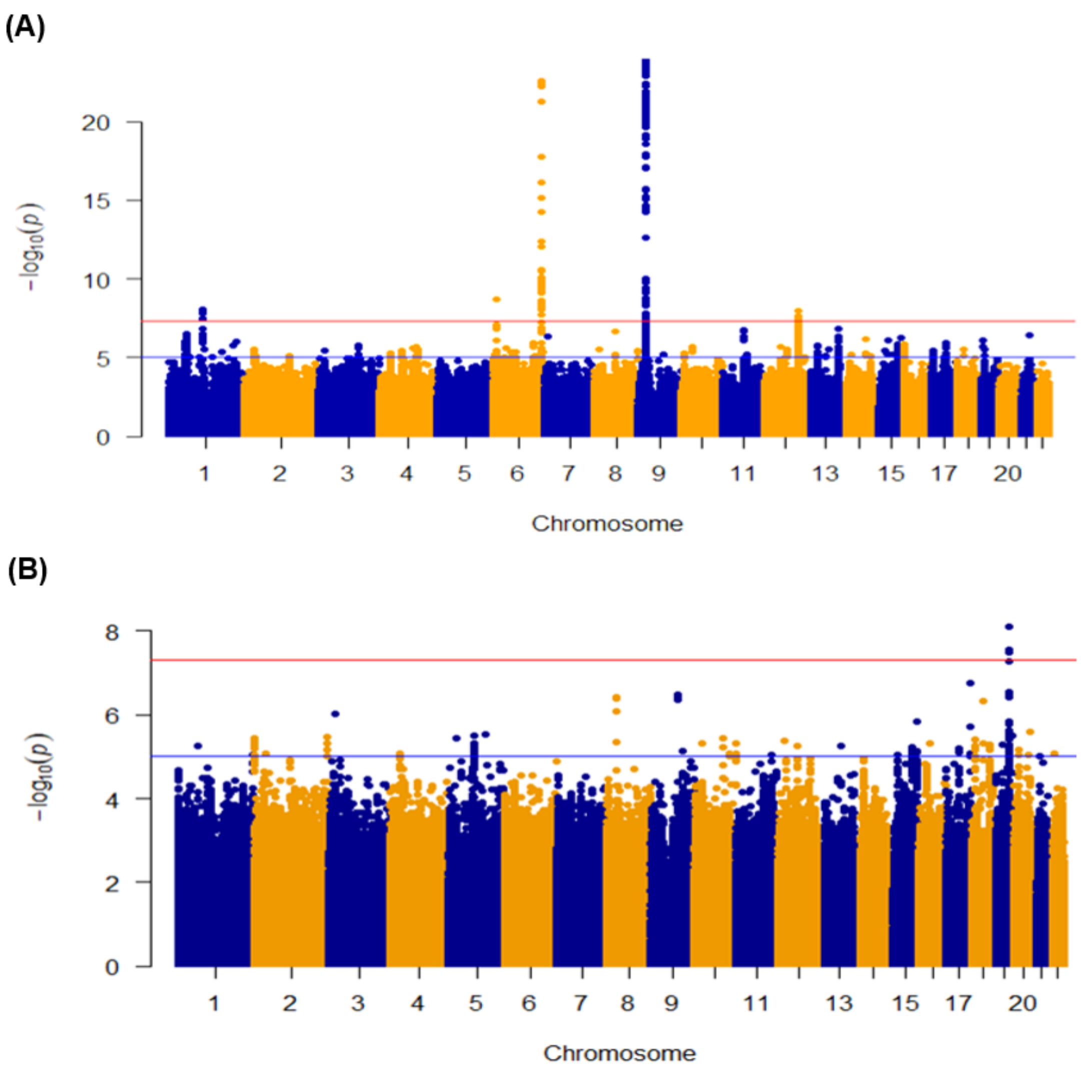

Supplementary Figure 3. Manhattan plots of GWAS for CV risk (A) and ACM (B) in UK Biobank

The SNV's associations with each outcome (negative logarithm of the P-value, y axis) are plotted against their chromosomal locations (x-axis). The red line represents the threshold for genome wide significance (*P* < 5 x 10^{-8}) while the blue line represents the lookup significance threshold (1 x 10^{-6}).

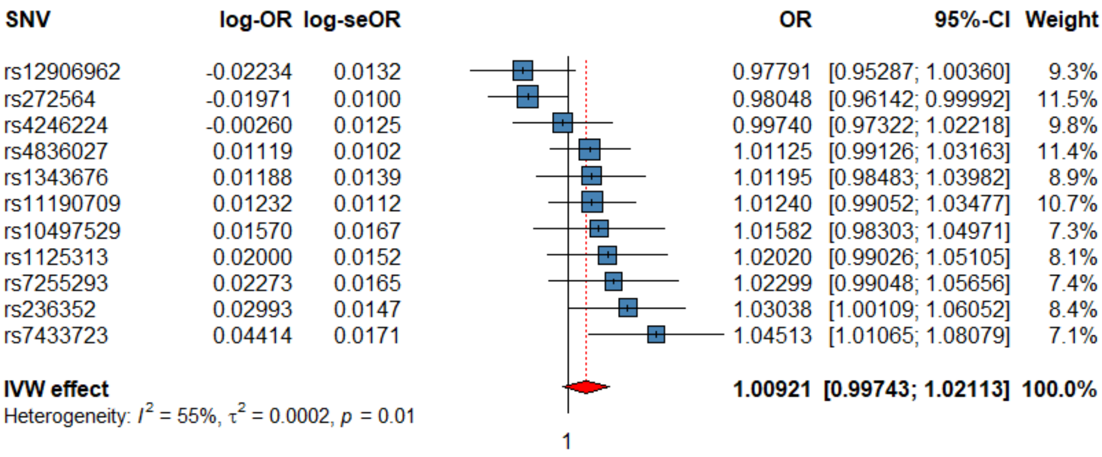

Supplementary Figure 4: Associations of 1 genetically predicted increase in beats per minute of HRI with CAD risk.

SNV: single-nucleotide polymorphism, OR: odds ratio, se: standard error, CI: confidence interval, I2: heterogeneity statistic I^2, τ^2: between-SNV variance.

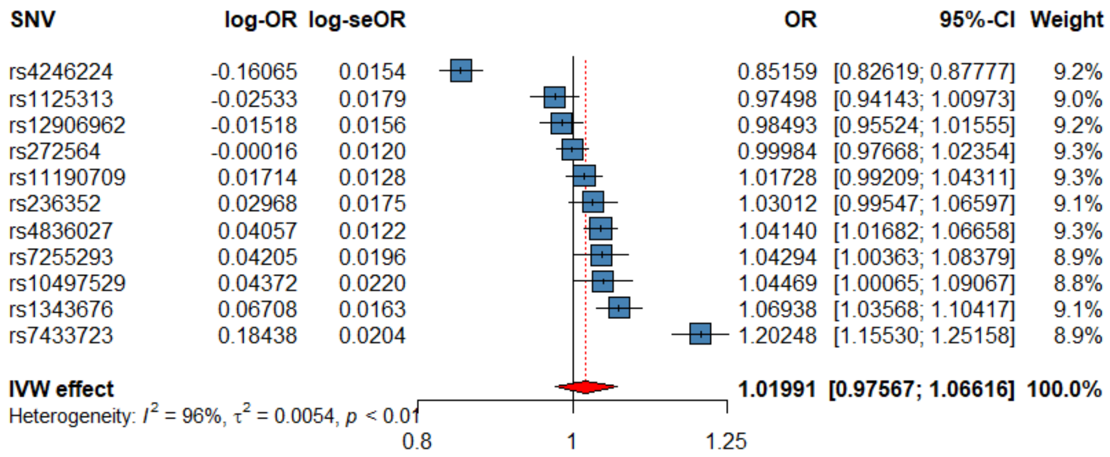

Supplementary Figure 5: Associations of 1 genetically predicted increase in beats per minute of HRI with AF risk.

SNV: single-nucleotide polymorphism, OR: odds ratio, se: standard error, CI: confidence interval, I2: heterogeneity statistic I^2, τ^2: between-SNV variance.

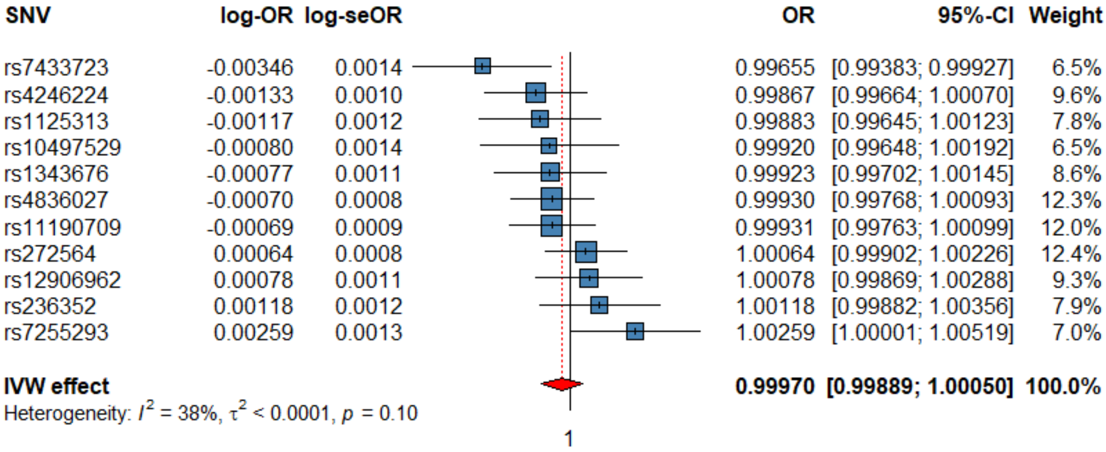

Supplementary Figure 6: Associations of 1 genetically predicted increase in beats per minute of HRI with ACM risk.

SNV: single-nucleotide polymorphism, OR: odds ratio, se: standard error, CI: confidence interval, I2: heterogeneity statistic I^2, τ^2: between-SNV variance.

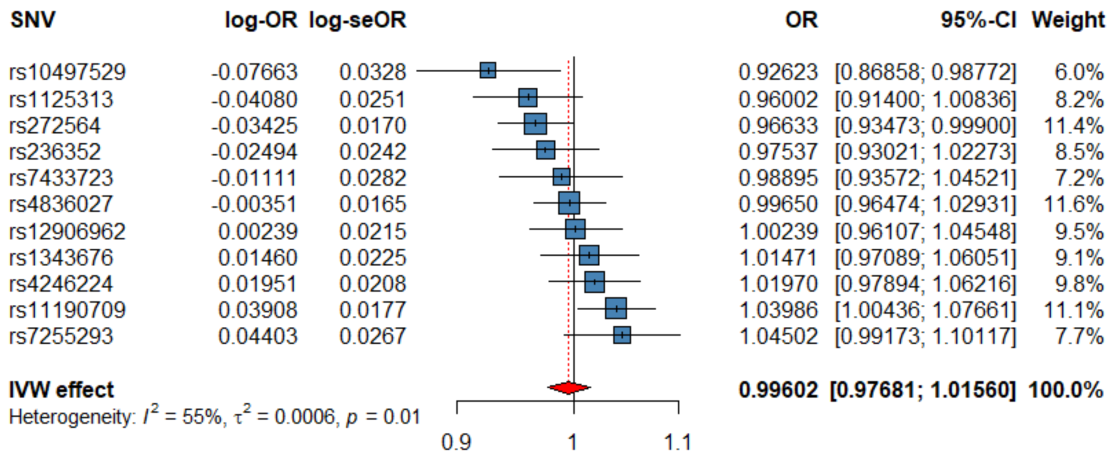

Supplementary Figure 7: Associations of 1 genetically predicted increase in beats per minute of HRI with IS risk.

SNV: single-nucleotide polymorphism, OR: odds ratio, se: standard error, CI: confidence interval, I2: heterogeneity statistic I^2, τ^2: between-SNV variance.

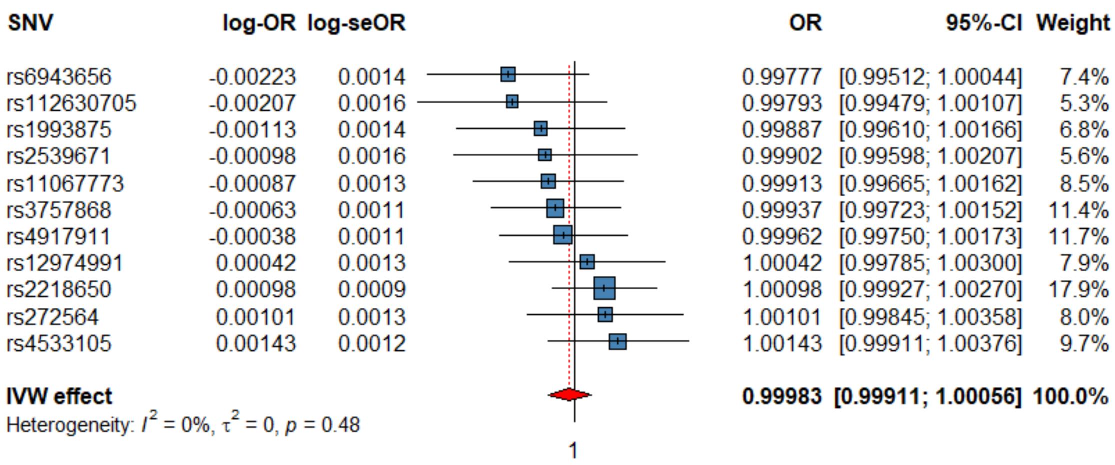

Supplementary Figure 8: Associations of 1 genetically predicted increase in beats per minute of HRR with ACM risk.

SNV: single-nucleotide polymorphism, OR: odds ratio, se: standard error, CI: confidence interval, I2: heterogeneity statistic I^2, τ^2: between-SNV variance.

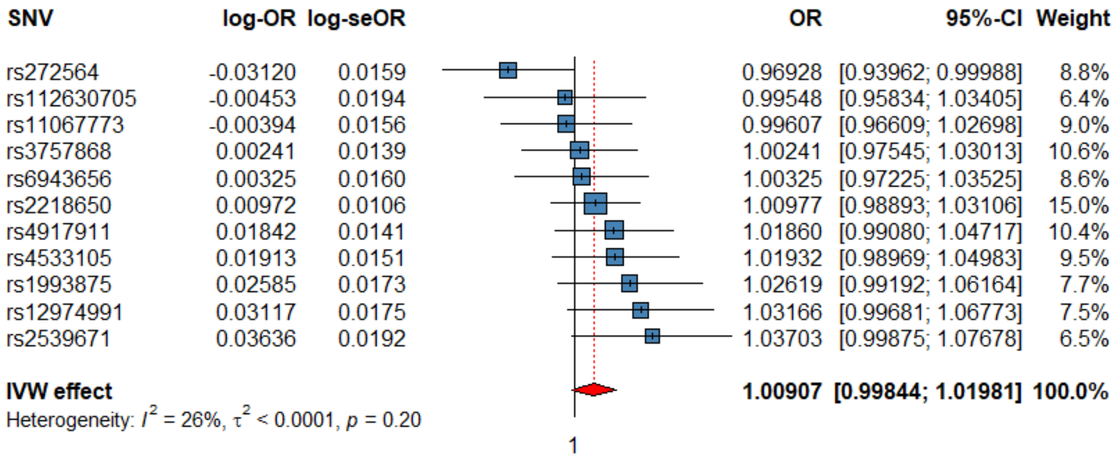

Supplementary Figure 9: Associations of 1 genetically predicted increase in beats per minute of HRR with CAD risk.

SNV: single-nucleotide polymorphism, OR: odds ratio, se: standard error, CI: confidence interval, I2: heterogeneity statistic I^2, τ^2: between-SNV variance.

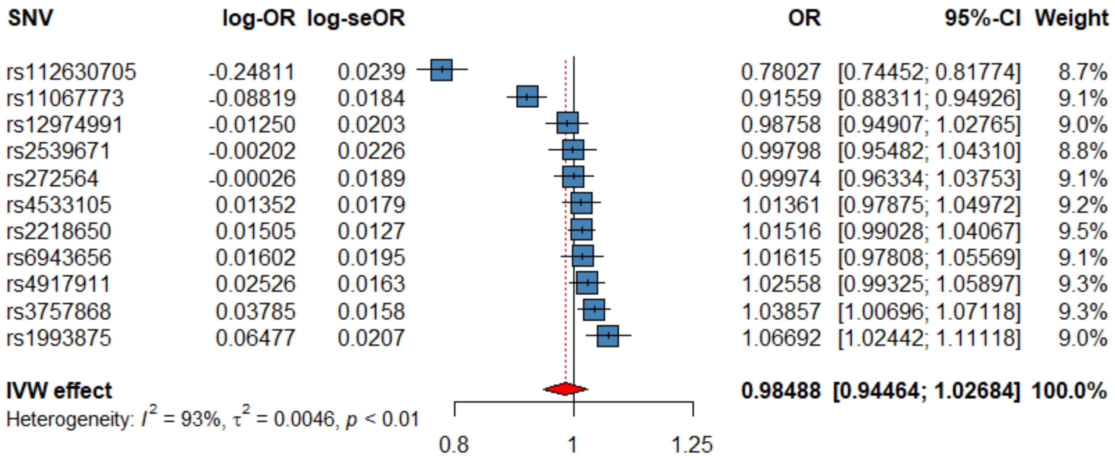

Supplementary Figure 10: Associations of 1 genetically predicted increase in beats per minute of HRR with IS risk.

SNV: single-nucleotide polymorphism, OR: odds ratio, se: standard error, CI: confidence interval, I2: heterogeneity statistic I^2, τ^2: between-SNV variance.

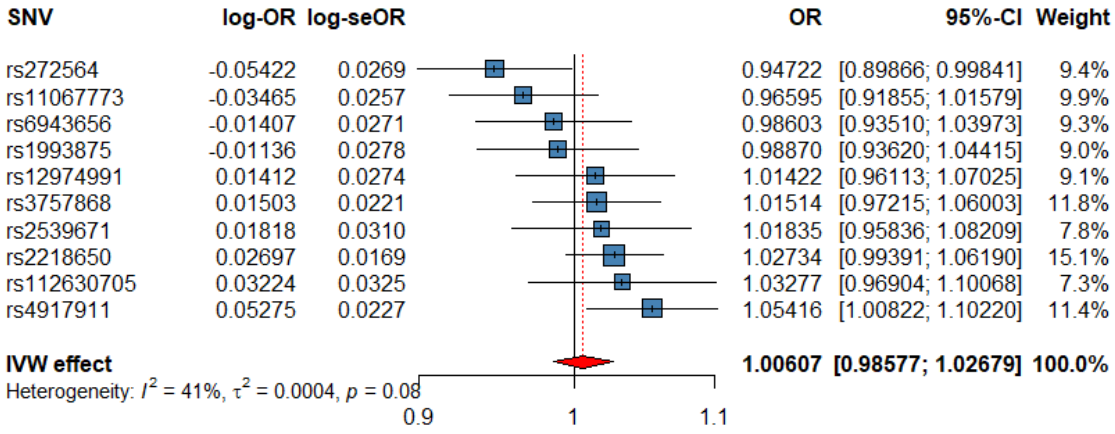

Supplementary Figure 11: Associations of 1 genetically predicted increase in beats per minute of HRR with AF risk.

SNV: single-nucleotide polymorphism, OR: odds ratio, se: standard error, CI: confidence interval, I2: heterogeneity statistic I^2, τ^2: between-SNV variance.

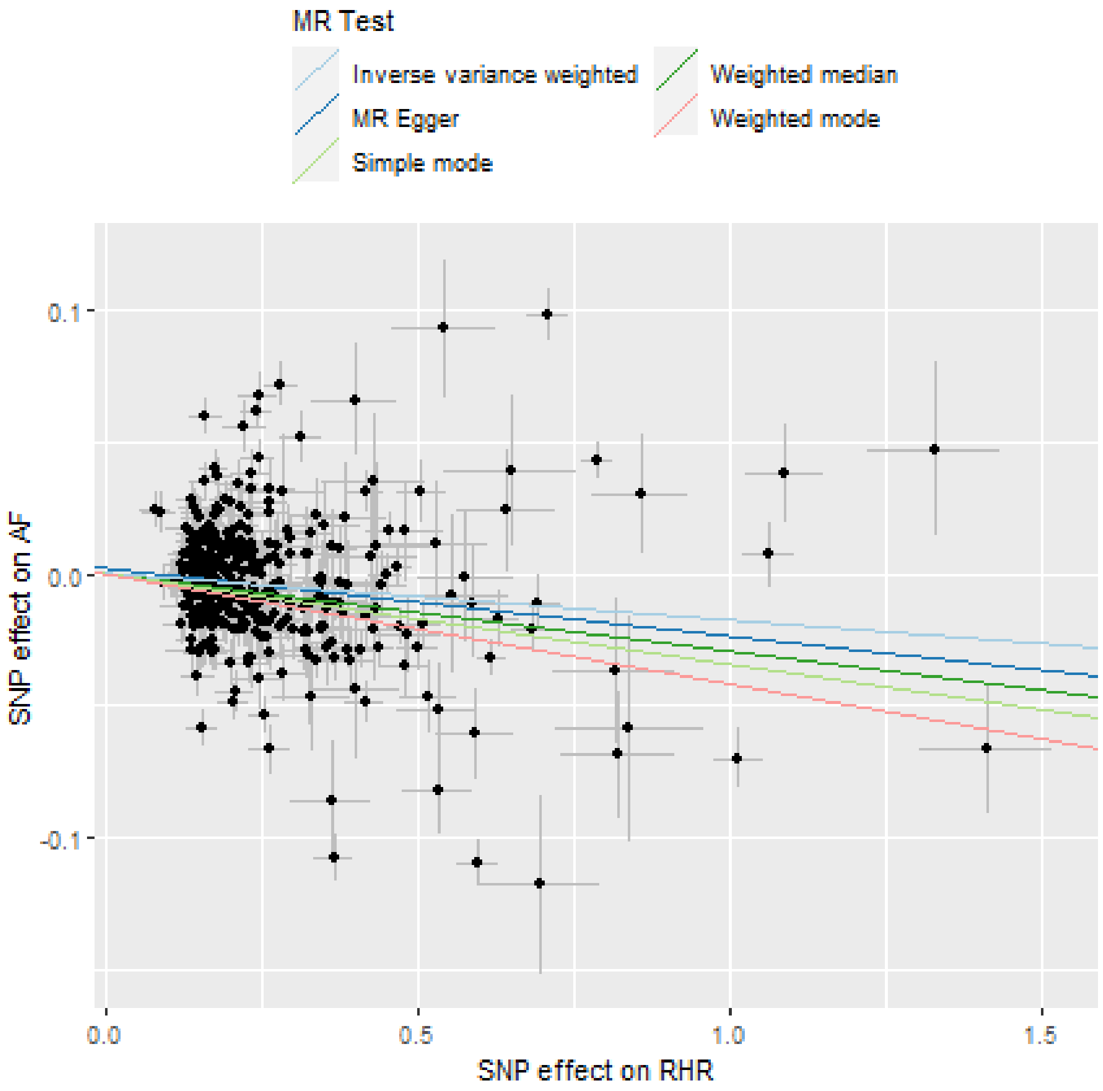

Supplementary Figure 12: Associations of 1 genetically predicted increase in beats per minute of resting HR with AF risk.

SNV: single-nucleotide polymorphism, AF: atrial fibrillation, RHR: resting heart rate.

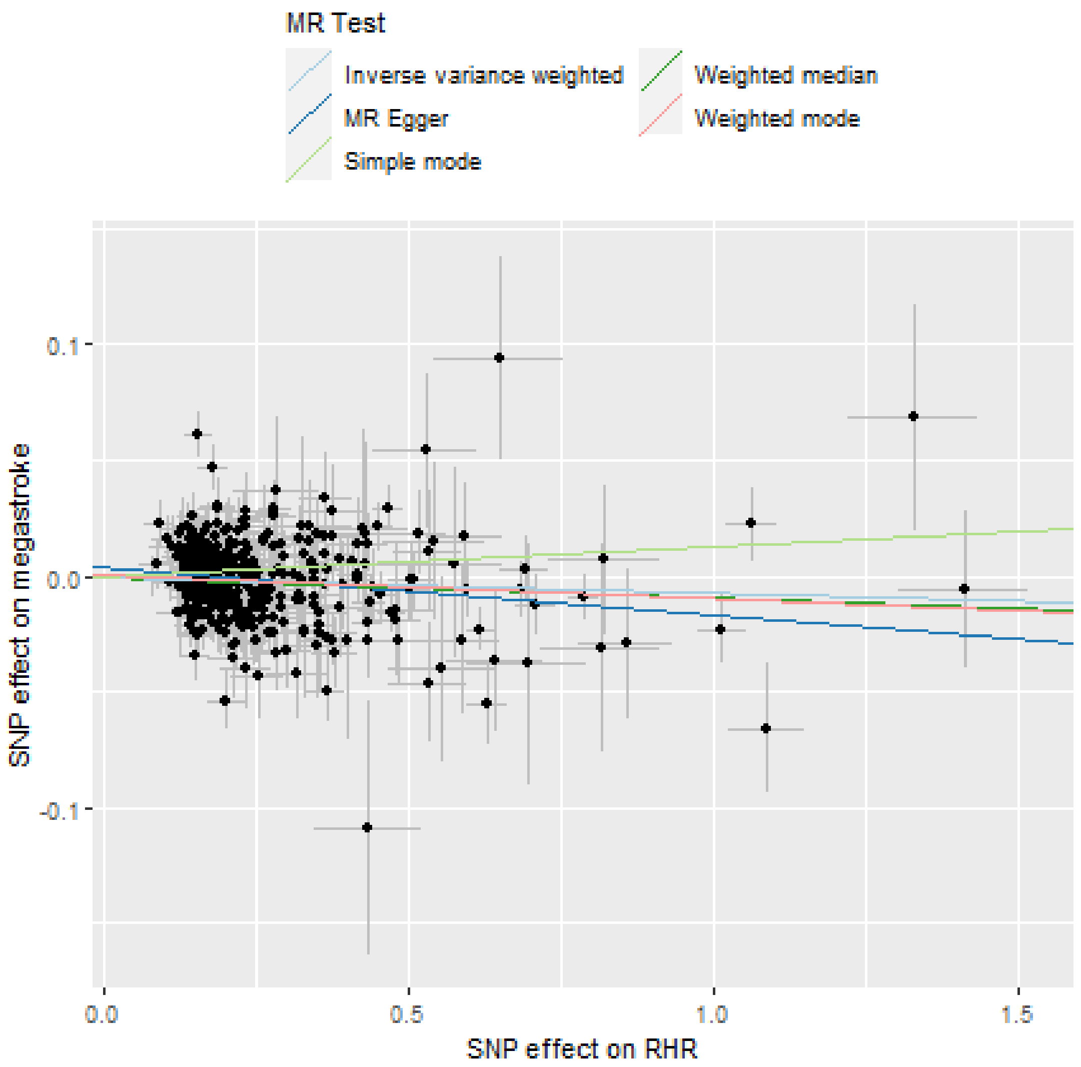

Supplementary Figure 13: Associations of 1 genetically predicted increase in beats per minute of resting HR with IS.

SNV: single-nucleotide polymorphism, RHR: resting heart rate.

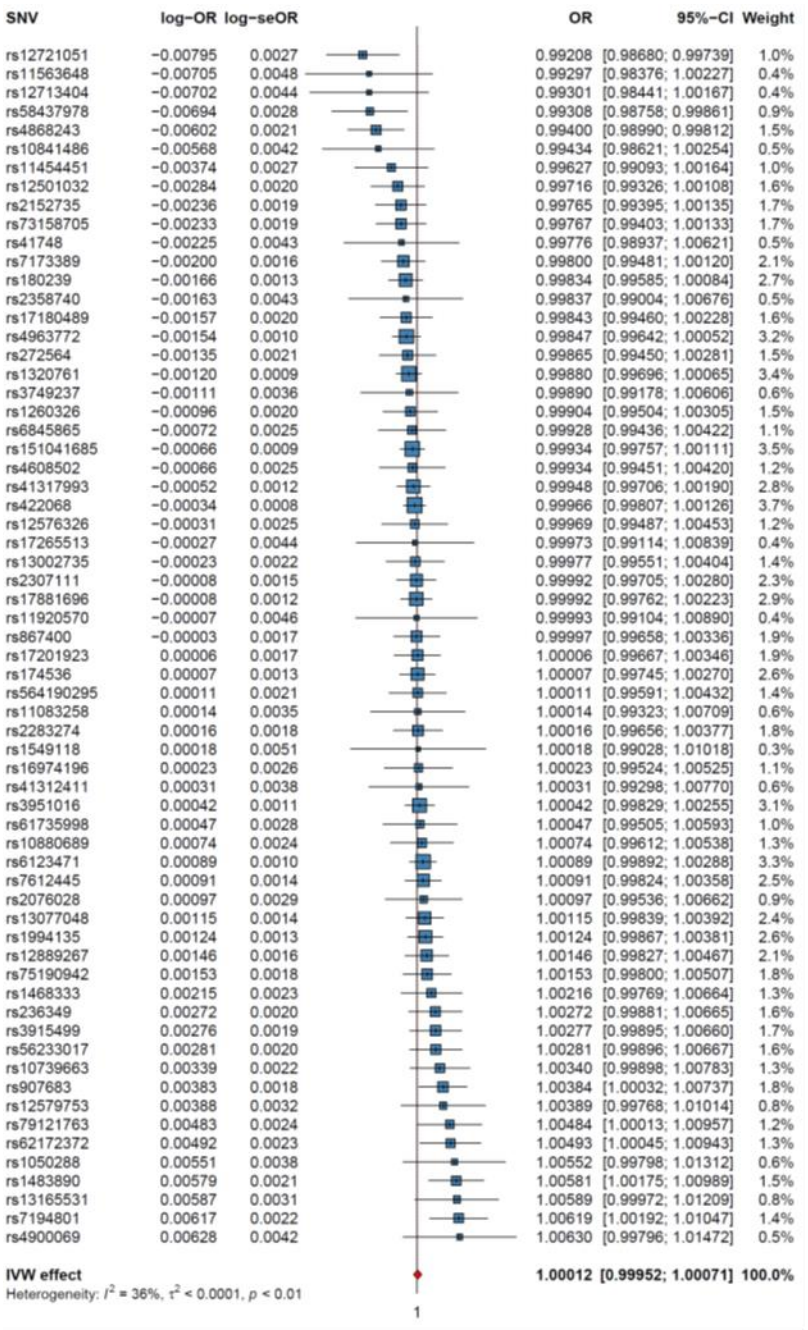

Supplementary Figure 14: Associations of 1 genetically predicted increase in beats per minute of resting HR with CV risk.

SNV: single-nucleotide polymorphism, OR: odds ratio, se: standard error, CI: confidence interval, I2: heterogeneity statistic I^2, τ^2: between-SNV variance.

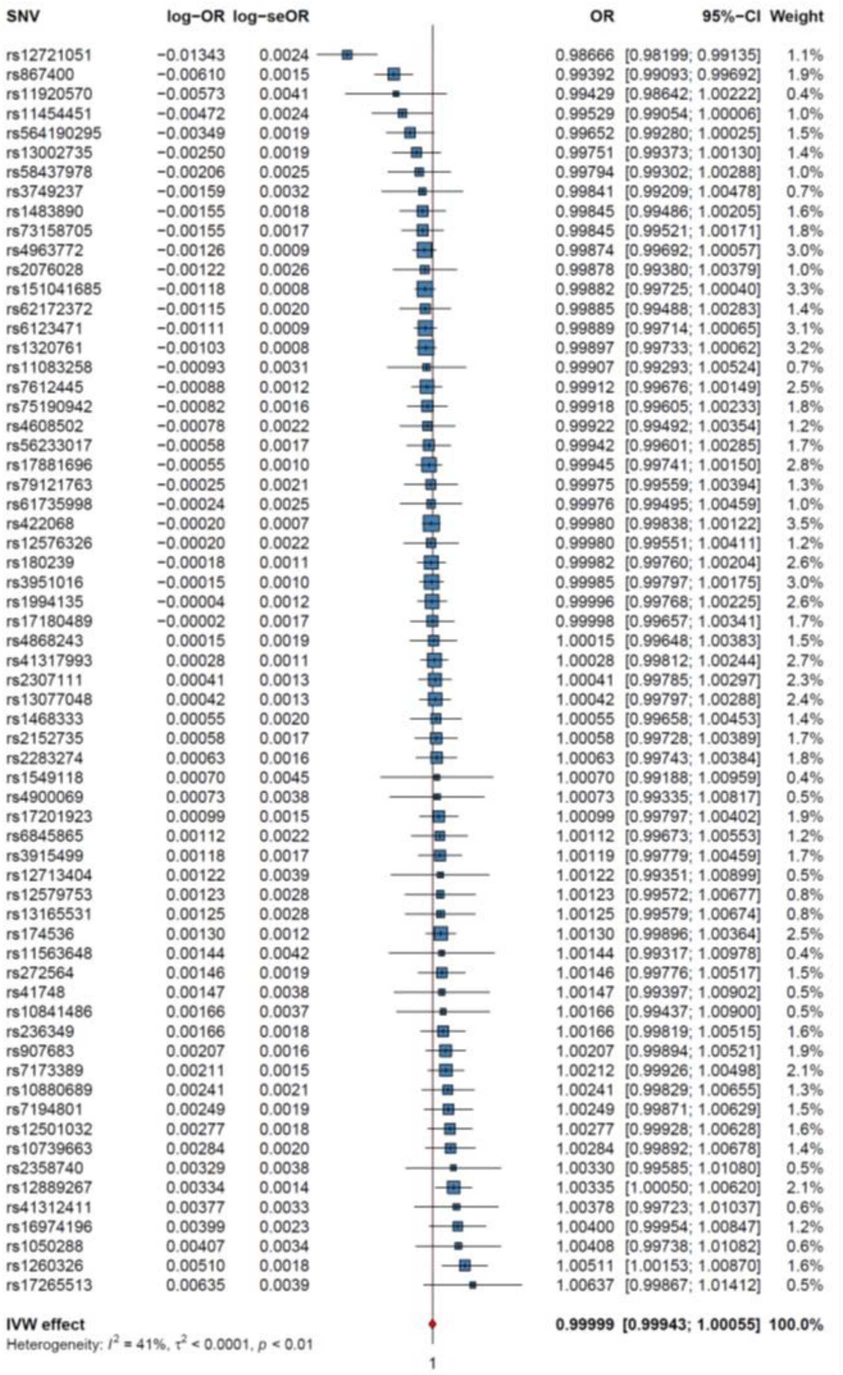

Supplementary Figure 15: Associations of 1 genetically predicted increase in beats per minute of resting HR with ACM risk.

SNV: single-nucleotide polymorphism, OR: odds ratio, se: standard error, CI: confidence interval, I2: heterogeneity statistic I^2, τ^2: between-SNV variance.

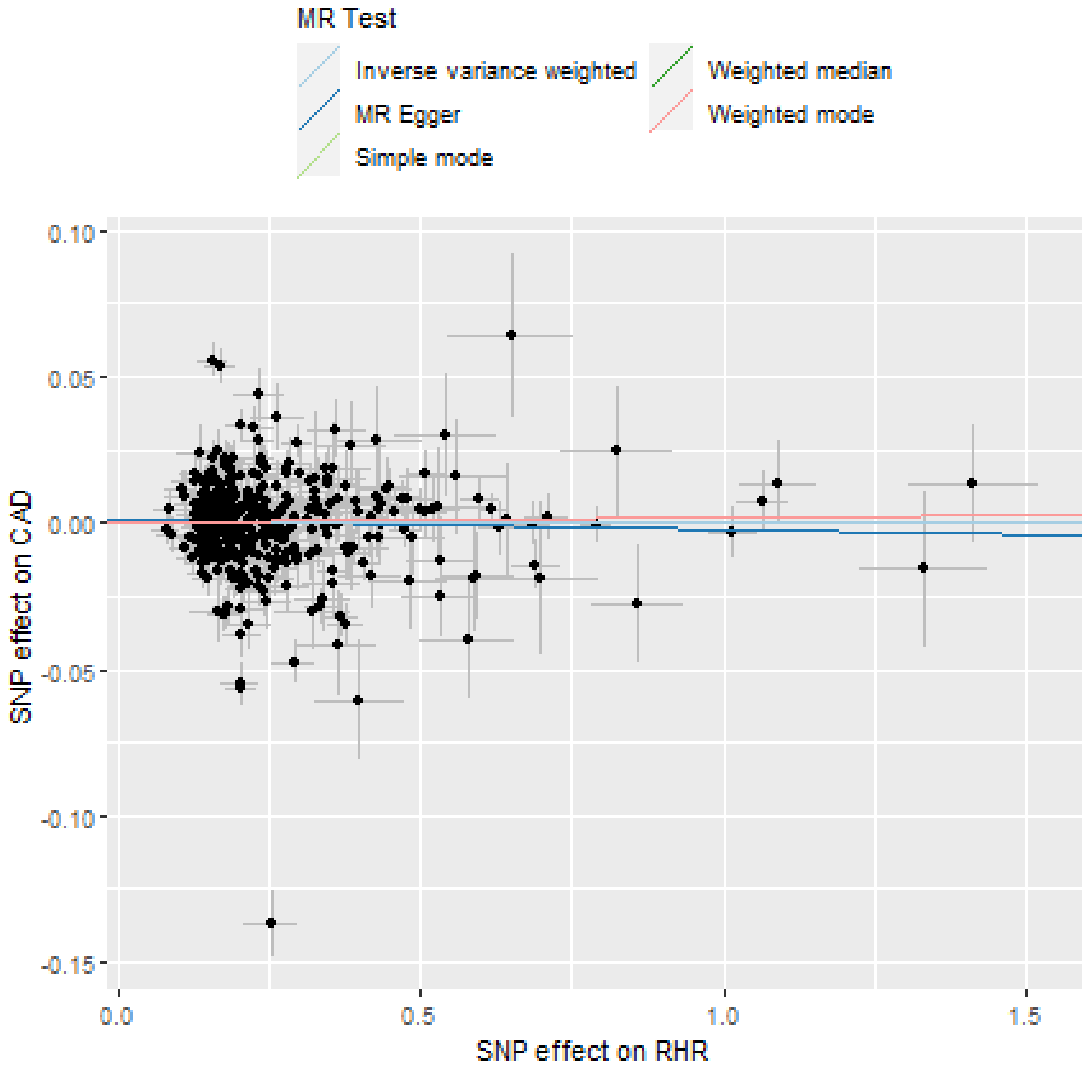

Supplementary Figure 16: Associations of 1 genetically predicted increase in beats per minute of resting HR with CAD risk.

SNV: single-nucleotide polymorphism, OR: odds ratio, se: standard error, CI: confidence interval, I2: heterogeneity statistic I^2, τ^2: between-SNV variance.
